# Cultivation of endogenous algal consortia for aquaculture wastewater remediation as a strategy toward net-zero carbon emission: from laboratory research to practical application

**DOI:** 10.3389/fnut.2025.1600232

**Published:** 2025-09-08

**Authors:** Limin Yang, Qian Lu

**Affiliations:** ^1^School of Life Sciences, Jiangsu University, Zhenjiang, China; ^2^School of Grain Science and Technology, Jiangsu University of Science and Technology, Zhenjiang, China

**Keywords:** aquaculture wastewater, carbon emission, algal consortia, nutrient removal, biomass

## Abstract

**Introduction:**

Endogenous algal consortia were cultivated in a recirculating aquaculture system to recover nutrients from wastewater and facilitate carbon sequestration.

**Methods:**

This study investigated the microbial community composition of the algal consortia, the roles of algae and associated microorganisms, the optimization of operational parameters, and the carbon emissions of the pilot-scale system.

**Results and discussion:**

The results showed that filamentous algae, particularly *Schizomeris* sp., are the dominant species in algal consortia. The interaction of algae and bacteria enhanced biomass production (0.90 g/L) and improved TOC removal efficiency (80.38%), demonstrating the excellent performance of algal consortia in wastewater treatment. In laboratory research, under optimal conditions, the carbon retention of algae-based aquaculture wastewater treatment reached 185.20 mg carbon/L of wastewater. In the 150-day pilot-scale experiment, 50.76 kg of carbon (feed) was input into the aquaculture system, while 11.50 kg of carbon was outputted as fish product, and algae biomass containing 39.27 kg of carbon was produced, resulting in an aquaculture process with net-zero carbon emission. The results of this study will provide a theoretical basis and practical strategies for the development of an eco-friendly aquaculture mode toward carbon neutrality.

## 1 Introduction

Aquaculture, which provides high-quality protein to humans and supports the livelihood of numerous people worldwide, is an important sector of the economy (1). In recent years, with the gradual depletion of offshore fishery resources, the development of aquaculture has become more and more important. It was predicted that the total fish supply will reach 186 million tons by 2030, and aquaculture will be entirely responsible for such a huge increase in fish production (1, 2). However, due to the excessive input of feed and the accumulation of fish feces, aquaculture, particularly aquaculture with high stocking density, produces a huge amount of organically-rich wastewater, placing a significant amount of pressure on environmental protection (1, 3).

As carbon neutrality by the middle century is a necessary condition for the achievement of Paris Agreement temperature goals, the carbon emission of aquaculture is becoming a hot research topic recently (4). It was estimated that 10.9 Tg carbon was generated from 39.9 Mt. of aquafeed by global aquaculture in 2016 (5). In 2017, global aquaculture contributed approximately 0.49% of anthropogenic greenhouse gas (GHG) emissions (6). A couple of recent studies analyzed the carbon footprints of different aquaculture models and fish species, confirming the intensive carbon emission of fish rearing (7, 8). Under this situation, the treatment of organic-rich aquaculture wastewater (AW) with the purpose of reducing carbon emission merits the attention of researchers.

Water remediation and recycling have been regarded as an applicable strategy to reduce the water consumption of intensive aquaculture and attenuate the negative effects of aquaculture activity on the environment (7). Chemical oxidation and anaerobic fermentation, both highly efficient degrading organics, are widely adopted in academic research and aquaculture practice; however,these methods are associated with significant carbon emissions. In recent years, aquaponics, which integrates hydroponics and aquaculture, has been developed to reduce the carbon emissions of aquaculture activity (9). However, the practical application of this model is challenged by a couple of problems, such as the low growth rate of vegetables, the perishability of fresh vegetable products, and the fluctuation of market demand (9, 10). Therefore, technologies and methods applied currently in aquaculture for AW treatment cannot fulfill the requirement for achieving the goal of carbon neutrality.

Algae are a group of microorganisms with the ability to assimilate atmospheric CO_2_ and organic carbon by photosynthetic and heterotrophic metabolisms, respectively. Theoretically, algae cultivated in wastewater could capture the atmospheric CO_2_, compensating for the carbon emissions occurring in wastewater treatment. In addition, algal cells can assimilate organic carbon in wastewater, thereby directly reducing carbon emissions from wastewater treatment. Recently, the employment of native algae for wastewater treatment has emerged into the limelight (11, 12). Compared to the commercial algae, native algae are more adaptable to the local environment, thus having much better performance in nutrient assimilation and biomass production. In addition to the algae screening, growth conditions, and harvesting techniques for biomass production and harvesting have been intensively studied as well (13, 14).

Herein, an innovative model that integrates algae culture with aquaculture is proposed to recover carbon from AW and reduce the carbon emission of aquaculture activity. Three questions to be answered by this work are listed as follows: (1) What is the fate of organic carbon in AW during the application of traditional treatment technology? (2) How to develop an algae-based AW treatment model for carbon recovery in an efficient and cost-saving way? (3) What is the performance of algal consortia in the pilot-scale aquaculture system for carbon emission reduction? It is expected that by addressing the aforementioned questions, we will make technological breakthroughs in recirculating aquaculture systems.

Innovative points of this study include the investigation of algae-based AW treatment from a carbon emission perspective and the extension of research scope to large-scale aquaculture systems rather than being confined to laboratory experiments. The advantages of this study over previous studies growing commercial microalgae or using traditional technologies for AW treatment are listed as follows: (1) The employment of endogenous algal consortia mainly consisted of filamentous algae for AW treatment simplified the biomass harvesting process; (2) Endogenous algal consortia, which are consisted of various bacteria and algae with synergistic relations, perform well in organics degradation and nutrient assimilation; (3) Attributed to the excellent photosynthetic performance of endogenous algal consortia, the integrated fish production and AW treatment become a net-zero carbon process, upgrading the aquaculture activity toward the goal of carbon neutrality.

## 2 Materials and methods

### 2.1 Experimental design

**Figure 1:**
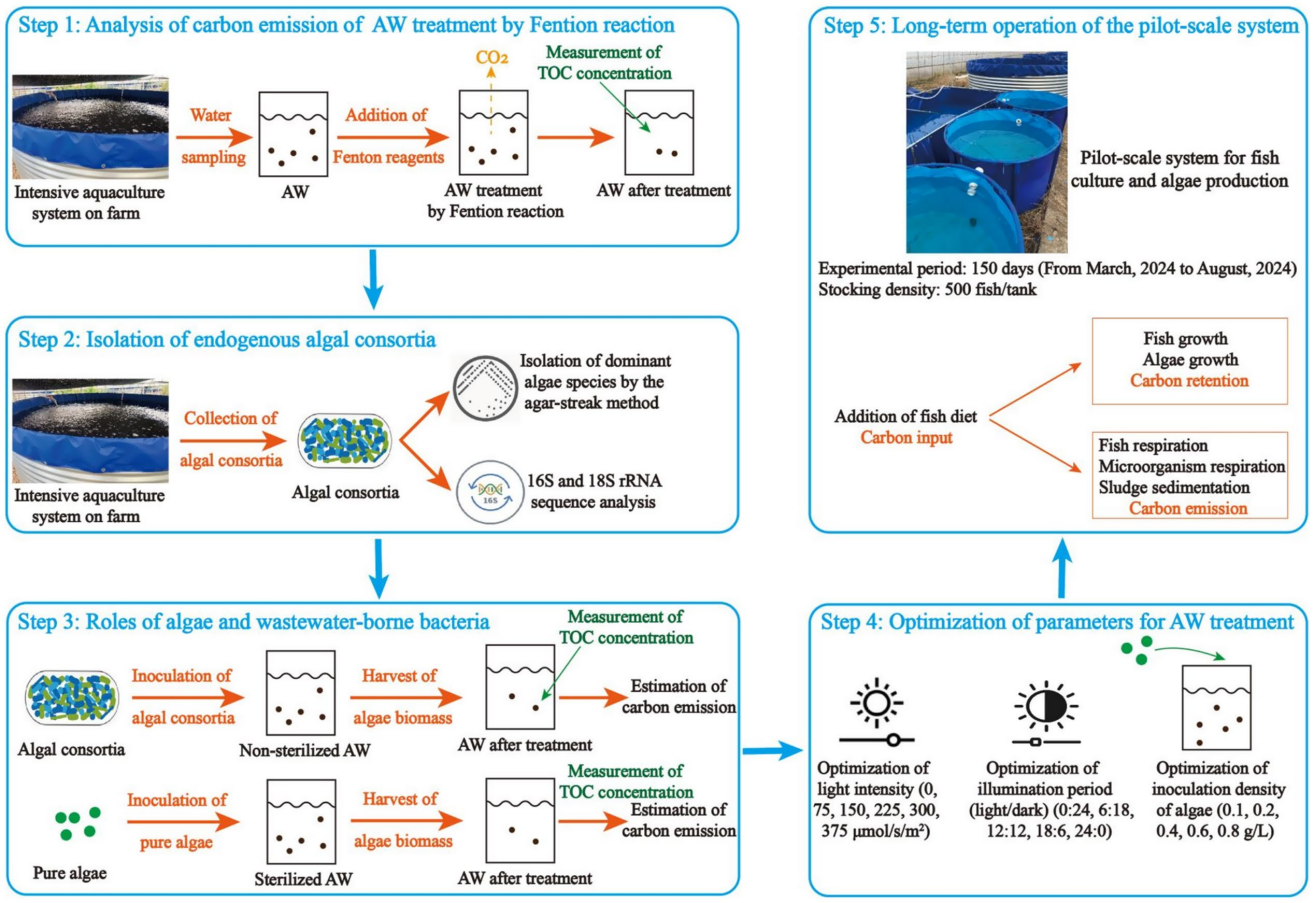
Experimental design.

This study, which aims to reduce the carbon emission in AW remediation and make the aquaculture activity environmentally-friendly, was carried out in five steps (Figure 1): (1) An analysis of carbon emission of AW treatment by Fenton oxidation, a traditional technology widely used for wastewater treatment, was conducted; (2) Isolation of endogenous algal consortia was conducted. At this step, the dominant species in algal consortia were isolated; (3) Roles of algae and wastewater-borne bacteria in AW remediation were identified. At this step, algal consortia and pure algae were inoculated in non-sterilized and sterilized AW, respectively, for nutrient recovery and the effects of algae growth on carbon emission of AW remediation were assessed in laboratory-scale experiment; (4) Some important parameters, including light intensity, illumination period, and inoculation ratio, were optimized to enhance the carbon sequestration in AW remediation. (5) Long-term operation of the pilot-scale system was conducted to perform the algae-based carbon recovery for AW remediation, and the carbon emission of the whole process in the pilot-scale experiment was estimated.

In this study, all the experiments and tests were conducted in triplicate, and the results were expressed as mean ± standard deviation.

### 2.2 Wastewater, fish, and algae

AW was obtained from a local aquaculture farm (Zhenjiang, China) in October 2023 and stored at 4°C before the laboratory-scale experiment. This farm cultured largemouth bass (*Micropterus salmoides*) in the intensive aquaculture systems at a high density (60 fish/m^3^), and AW was collected from the outlets of the systems.

The fish used in this study is the largemouth bass, and juvenile fish were purchased from Jiangsu Shuaifeng Group Co., Ltd. (Nanjing, China). The juvenile fish were temporarily placed in a pool for 10 days and then transferred to recirculating aquaculture systems for the experiment. The pelleted feeds for fish growth were also purchased from Jiangsu Shuaifeng Group Co., Ltd. (Nanjing, China).

In the long-term operation of the recirculating aquaculture system, due to the accumulation of fish feces and the eutrophication of water, a large amount of algal consortia with high stickiness were present on the surface of the side wall of the fish-rearing tank (Supplementary Figure S1a). To identify the dominant species, algal consortia were collected and then sent to Weiji Biotechnology (Shanghai) Ltd. for 16S and 18S rRNA sequence analysis. The isolated algal consortia were preserved in artificial BG11 medium, of which the nutrient profile was documented by a previous study (15), in 250 mL Erlenmeyer flasks. Fluorescent lamps were employed to provide illumination for algae, and light intensity was controlled at approximately 150 μmol/m^2^/s. Algae harvested from the artificial medium by centrifugation and then inoculated in AW for wastewater treatment.

### 2.3 Experimental procedure

#### 2.3.1 Traditional technology for AW remediation

In this study, Fenton oxidation, which can efficiently convert organic carbon to CO_2_ by oxidation, was employed as a traditional technology for AW remediation (16). Hydrogen peroxide (H_2_O_2_) and ferrous sulfate (FeSO_4_) were added in AW at a concentration of 4 mM/L and 1 mM/L, respectively. The initial pH value was adjusted to 4.5, and the treatment period was set as 240 min. By the end of the reaction, the concentration of TOC in the AW was measured to estimate the carbon fate.

#### 2.3.2 Collection of endogenous algal consortia

The dominant species, *Schizomeris* sp., were isolated from the endogenous algal consortia collected from the wall surface of a traditional fish-rearing tank by using the agar-streak method. In this study, pure *Schizomeris* sp. and algal consortia were employed to treat AW individually in the following experiment. In addition, the water obtained from the fish-rearing tank was also subjected to the 16S and 18S rRNA sequence analysis (Taihe Biotechnology Co. Ltd., China).

#### 2.3.3 Algae cultivation for AW remediation

*Schizomeris* sp. and algal consortia were inoculated in the sterilized AW and non-sterilized AW, respectively, for nutrient recovery. In this study, a light-emitting diode (LED) was employed to provide consistent illumination to algae. Light intensity, illumination period (light: dark), room temperature, and inoculation density of algae were set as 150 μmol/m^2^/s, 12 h:12 h, 25°C, and 0.2 g/L, respectively. In the 10-day period, biomass yield of algae and concentrations of TOC, TN, TP, and TAN were measured every 2 days. By the end of AW treatment, algae were harvested by membrane filtration and then subjected to the quantification of major compositions (crude protein, crude lipid, and carbon element). Carbon emission and carbon retention of algae-based AW remediation were calculated according to Equations 1, 2, respectively. Carbon recovery efficiency was calculated according to Equation 3.


(1) $$\mathrm{CE}=C_{\mathrm{TOC}-i}-{\mathrm{BY}}_{M}\times P_{C}$$

(2) $$\mathrm{CR}={\mathrm{BY}}_{M}\times P_{C}$$

(3) $$R_{C}=\frac{{\mathrm{BY}}_{M}\times P_{C}}{C_{\mathrm{TOC}-i}}\times 100\%$$

where CE and CR are short for carbon emission (mg carbon/L wastewater) and carbon retention (mg carbon/L wastewater), respectively; *R_C_* is carbon recovery efficiency (%); *C_TOC-i_* refers to the initial concentration of TOC in AW; *BY_M_* is the biomass yield of algae in AW remediation and *P_C_* is the percentage of carbon element in the dried algae biomass.

#### 2.3.4 Optimization of parameters for algae-based AW remediation

In a laboratory-scale experiment, a single-factor experiment was conducted to assess the effects of light intensity, illumination period, and inoculation density on biomass production and carbon emission during algae-based AW remediation. The actual levels of each independent variable are presented in Supplementary Table S1. Based on the experimental results, in this experiment, AW was not sterilized before the inoculation of algal consortia to create an environment with co-cultured algae and wastewater-borne bacteria. In this study, the optimal condition for algae-based AW treatment was identified according to the carbon recovery and biomass yield. Then, the carbon emission of algae-based AW remediation under the optimal condition was estimated accordingly.

#### 2.3.5 Long-term operation of the pilot-scale aquaculture system

**Figure 2:**
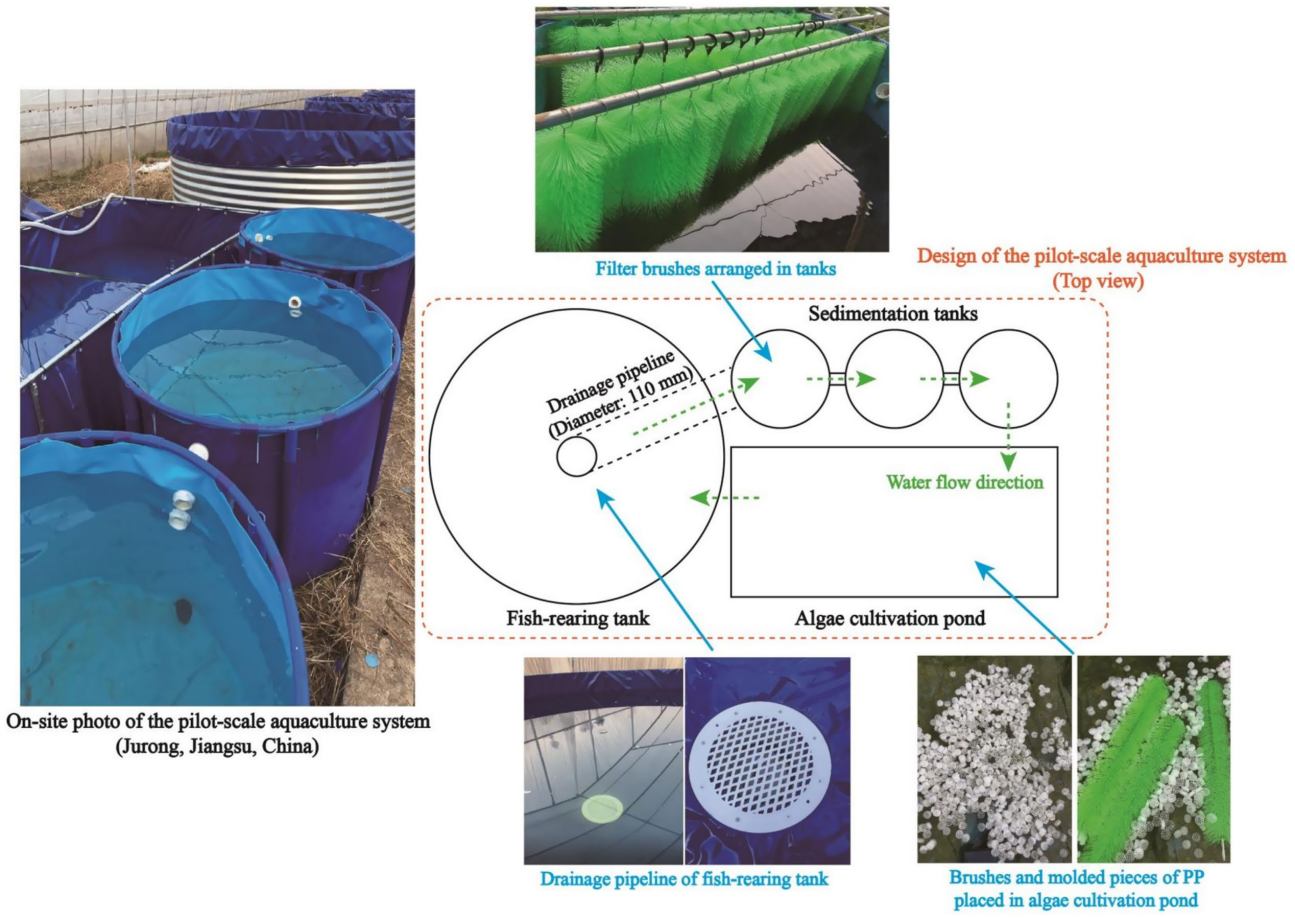
Layout of the pilot-scale system for algae-based aquaculture wastewater remediation.

The pilot-scale aquaculture system, of which the structure is presented in Figure 2, was employed to evaluate the practical applicability of algae-based AW remediation. Generally, the whole system consists of three major parts, namely a fish-rearing tank (Diameter: 3.0 m; Height: 1.2 m), three sedimentation tanks (Diameter: 1.2 m; Height: 0.8 m), and an algae cultivation pond (Length: 4.6 m; Width: 2.2 m; Height: 0.6 m). Air was pumped into the fish-rearing tank via aeration devices to maintain the DO content in a range of 4.0–5.5 mg/L. Filter brushes, which can provide attachment sites for the growth of bacteria, were arranged in sedimentation tanks to accelerate the degradation of organics in AW. In an algae cultivation pond, brushes and molded pieces of polypropylene (PP) were employed to provide attachment sites for algal consortia, and a circulation pump was operated to promote the water circulation. As shown in Figure 2, AW from the fish-rearing tank entered the sedimentation tanks via the drainage pipeline, and AW after the algae-based treatment was recirculated to the fish-rearing tank by a pump. Solar-cell power supply system was adopted to support the operation of the whole pilot-scale aquaculture system.

In this study, the pilot-scale experiment (150 days) lasted from March 2024 to August 2024. At the beginning of the 150-day experimental period, 500 largemouth basses (*Micropterus salmoides*) (Length: 5.0–6.0 cm; Weight: 5.0–7.0 g) were added to the fish-rearing tank. The fish were fed commercial fish feed two times each day. In this study, the commercial feed is enriched with crude protein (42.80%) and contains a high content of carbon (51.20%). The average amount of feed added daily for the fish rearing tank is shown in Supplementary Table S2. It should be noted that to meet the requirement of fish growth on nutrients and prevent the serious pollution caused by the excessive addition of fish feed, the amount of feed added was adjusted every 10 days according to the weight of the fish.

During the pilot-scale experiment, approximately 80% of the water in the fish-rearing tank was pumped into algae cultivation ponds for nutrient recovery every week. The retention time of the AW in algae cultivation ponds was set as 10 days. By the end of algae-based AW treatment, algae biomass was harvested every 10 days (Supplementary Figure S1b) and water was recycled to the fish-rearing tank. The harvested biomass was dehydrated and weighed. Then, the major compositions, including crude protein, crude lipid, and carbon element, of algae biomass were quantified. Carbon flux in the long-term operation of the pilot-scale aquaculture system was estimated accordingly. During the continuous operation, concentrations of TOC in the fish-rearing tank and algae cultivation pond were measured every 5 days.

### 2.4 Analytical methods

#### 2.4.1 Biochemical properties of wastewater

A portable pH analyzer was employed to identify the pH value of AW. The content of dissolved oxygen (DO) in AW was measured using the portable DO analyzer (Smart Sensor, China).

Concentrations of TOC, TN, TP, and TAN in AW were measured using assay kits (Hatch, USA) according to the operational manual for specific procedures. Nutrient removal efficiency is calculated according to Equation (4).


(4) $$\mathrm{NRE}=\frac{N_{i}-N_{f}}{N_{i}}\times 100\%$$

where NRE is short for nutrient removal efficiency (%); *N_i_* refers to the initial concentration of nutrient (mg/L); and *N_f_* refers to the final concentration of nutrient (mg/L).

#### 2.4.2 Algae biomass yield and composition analysis

Algae grown in artificial medium or AW were harvested by centrifugation (5,000 rpm and 10 min). The harvested biomass was dried in an oven to test the total volatile suspended solids (TVSS) according to the published method (17). In this study, TVSS was used to reflect the biomass yield of algae.

The dried biomass was subjected to the quantification of carbon and nitrogen elements by using the elemental analyzer (ThermoFisher, USA). Then, the content of crude protein in algae biomass was calculated based on the nitrogen content. In this study, the conversion factor of nitrogen-to-protein was set as 6.25 according to the previous study (17).

#### 2.4.3 Analysis of fish growth, feed utilization, and survival ratio

During the 150-day pilot-scale experiment, data were recorded for the analysis of fish growth, feed utilization, and survival ratio. Individual weight (g) and length (cm) were measured by randomly collecting 20 fish from the fish-rearing tank. The important parameters are calculated according to the specific methods documented in the previous study (18). By the end of the experiment, 20 fish were collected from the fish-rearing tank randomly and then subjected to the analysis of crude protein content and crude lipid content.

## 3 Results and discussion

### 3.1 Carbon emission in AW remediation by traditional technology

As shown in Table 1, AW obtained from the aquaculture farm contains a high concentration of TOC, reaching 238.50 mg/L. Hence, AW can be regarded as a carbon source with the potential of causing intensive carbon emission. Theoretically, if all of the organic carbon in AW is decomposed and released as CO_2_, 1 m^3^ AW will produce 0.875 kg CO_2_, which is equal to 0.442 m^3^ CO_2_ (the density of CO_2_ is set as 1.978 g/L). It should be noted that organic carbon in AW exists not only in the form of dissolved organics, but also in the form of suspended solids, of which the content is around 0.38 g/L in the wastewater (Table 1).

**Table 1:** Biochemical properties of aquaculture wastewater.

Item	Value
TOC	238.50 mg/L
TAN	2.62 mg/L
TN	21.44 mg/L
TP	12.91 mg/L
pH	6.69
SS	0.38 g/L
DO	5.26 mg/L

With the addition of Fenton reagents in AW, fast removal of TOC was observed. By the end of the treatment process, 94.63% of TOC in AW was removed, and the concentration of TOC in AW dropped to 12.80 mg/L (Supplementary Figure S2). In addition, the addition of Fenton reagents promoted the decomposition of suspended solids in wastewater, reducing the content of SS from 0.38 g/L to 0.07 g/L. The high removal efficiency of TOC is attributed to the superoxidative ability of the Fenton system (16). However, through the Fenton reaction, the majority of the removed organic carbon in AW was converted to CO_2_, making the AW treatment a positive carbon emission process. Therefore, although traditional technologies based on chemical oxidation could effectively remove the organic carbon in AW, potential threats of the intensive carbon emission to the environment merit the attention of researchers (19, 20).

### 3.2 Microbial community of endogenous algal consortia

**Figure 3:**
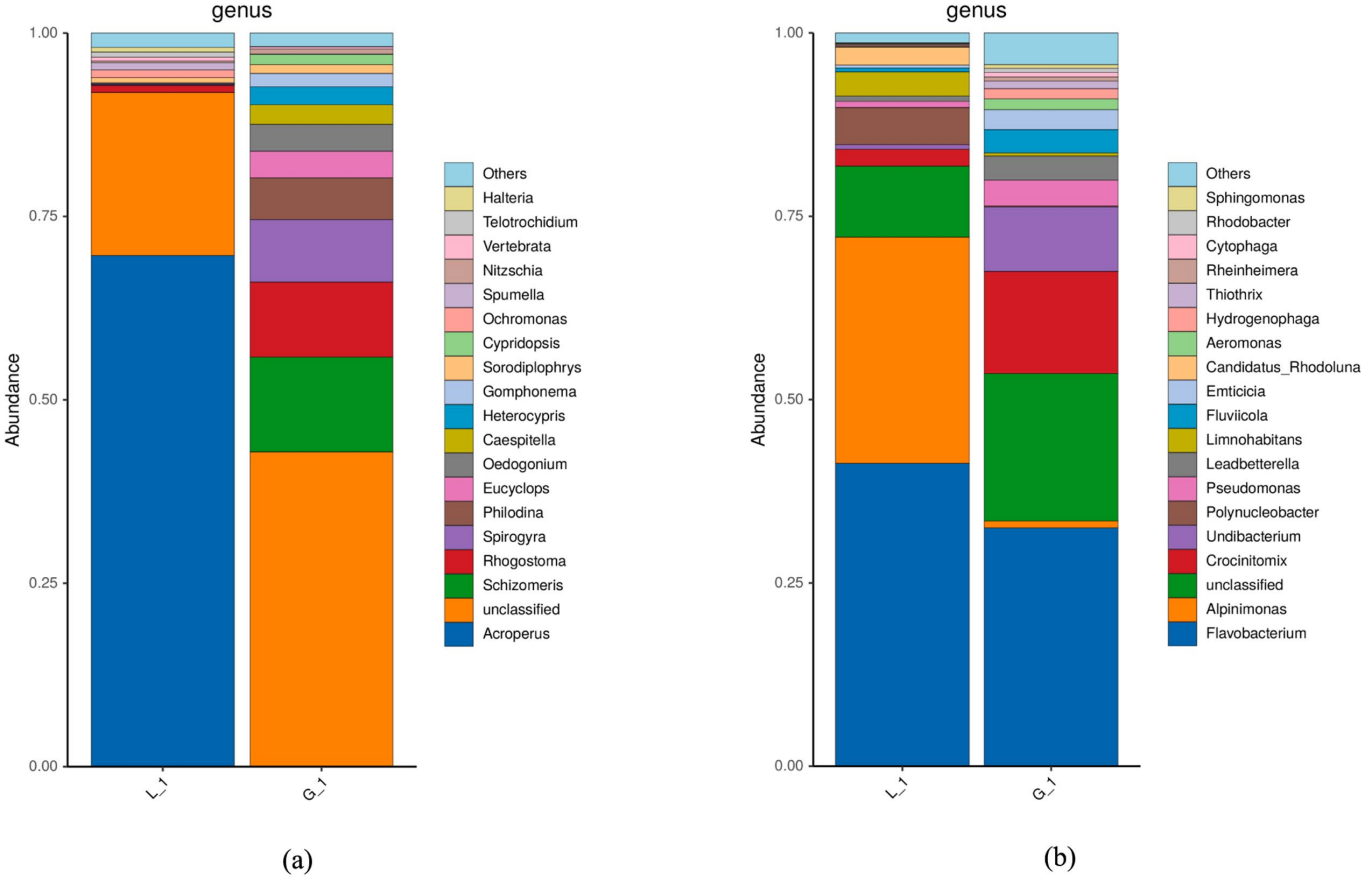
Microbial community of algal consortia and water collected from fish-rearing tank **(a)** Result of 18S rRNA sequence analysis; **(b)** Result of 16S rRNA sequence analysis (L_1: Water sample; G_1: Algal consortia sample).

The results of genetic sequence analysis show that the endogenous algal consortia were enriched with a variety of organisms, including algae, bacteria, and protozoa (Figure 3). Green algae, including *Schizomeris* sp., *Spirogyra* sp., *Oedogonium* sp., and *Caespitella* sp., were the dominant genera in algal consortia. However, in the water sample obtained from the fish-rearing tank, the abundances of green algae were much lower (Figure 3a). The main reason for this phenomenon is that algal consortia, which mainly consisted of filamentous algae, were tightly attached to the side wall of the fish-rearing tank, and the disturbance of water flow did not cause the washout of algae (Supplementary Figure S1a). In fact, due to the excellent performance of algae in nutrient assimilation, algae-based AW treatment has been intensively studied in previous publications (1). In previous studies, algal species widely adopted for AW treatment include *Chlorella* sp. and *Scenedesmus* sp. (Table 2), but the attachment ability of these algae with spherical cell structure is not well studied. As a result, the washout of algae occurs when the fluid shear force of water is high (13). If these spherical algae are cultivated in a recirculating aquaculture system for AW treatment, they will flow into the fish-rearing tank from the algae cultivation pond, resulting in the low visibility of the water in the fish-rearing tank and negatively impacting fish growth. In addition, some commercial algal species are not suitable to be used in the recirculating aquaculture system, although they may perform well in nutrient assimilation during AW treatment. For example, *Spirulina* sp. could remove around 100% ammonia, 50% nitrate, and 50% phosphorus in AW (21). However, the growth of *Spirulina* sp. can increase the pH of water dramatically, and the alkaline environment may result in the failure of fish culture (1). Compared with the commercial algae, endogenous algae have obvious advantages in following aspects: (1) endogenous algal consortia have developed stable physical and biological structure, which can effectively prevent the washout of algal cells; (2) endogenous algal consortia obtained from aquaculture system have no negative effect on water quality and fish growth; (3) endogenous algal consortia which have adapted to the aquaculture environment is supposed to have better performance in nutrient assimilation during AW treatment.

**Table 2:** Application of commercial algal species for aquaculture wastewater treatment.

Algal species	Source of aquaculture wastewater	Nutrient removal			Biomass yield (g/L)	Reference
		TN	TP	COD		
*Chlorella sorokiniana*	A containerized, high-density aquaculture system	86.42%	77.84%	TOC: 82.27%	1.93	(43)
*Chlorella pyrenoidosa*	Simulated aquaculture wastewater	96.96%	85.15%	88.53%	1.0–1.3	(44)
*Scenedesmus obliquus*	A seafood nursery company	100%	100%	90.6%	~2.25	(45)
*Nannochloropsis salina*	A seafood nursery company	100%	100%	71.8%	~0.70	(45)
*Chlorella vulgaris*	A seafood nursery company	100%	100%	86.6%	3.22	(45)
*Chlorella vulgaris*	A trout farm aquaculture facility	93.5%	92.7%	/	0.4559	(46)
*Scenedesmus obliquus*	An aquaculture research facility	68.09%	~100%	42%	1.25	(47)
*Chlorella sorokiniana*	An aquaculture research facility	67.89%	~100%	69%	1.51	(47)
*Ankistrodesmus falcatus*	An aquaculture research facility	75.29%	98.52%	61%	2.25	(47)
*Tetraselmis* sp.	Synthetic marine aquaculture wastewater	95.5%	94.4%	61.4%	1.19	(48)

**Figure 4:**
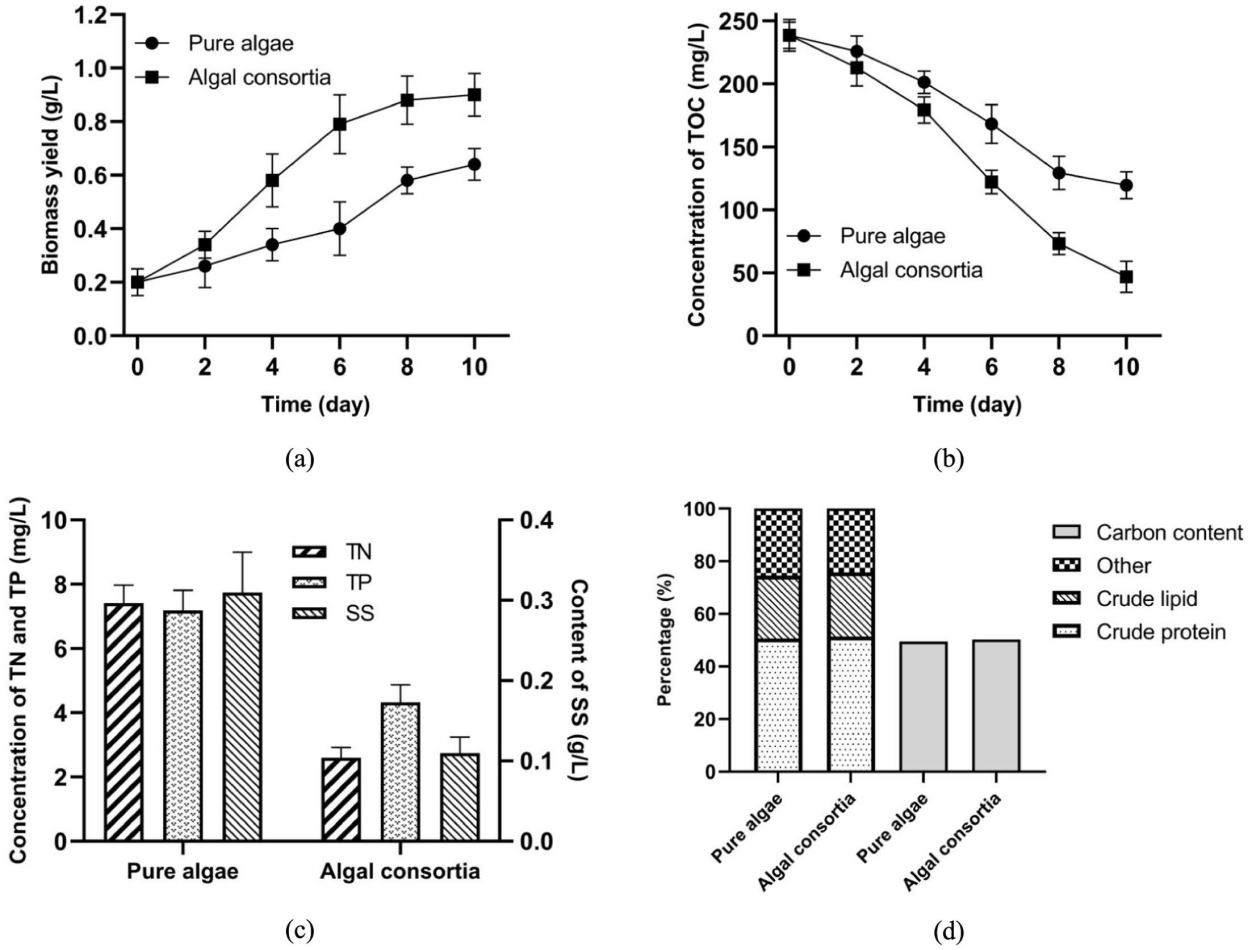
Effects of algae cultivation on the biochemical properties of AW **(a)** Biomass yield, **(b)** Removal of TOC, **(c)** Removal of TN, TP, and SS; **(d)** Major compositions of the harvested biomass.

In this study, it was discovered that algal consortia had higher biomass yield and removed more organic carbon in AW than the isolated pure algae (Figures 4a,b). Such an advantage is mainly attributed to the synergistic cooperation between algae and bacteria in algal consortia. According to the analysis of microbial structure, dominant microorganisms in algal consortia can be classified into two categories, namely algae (*Schizomeris* sp., *Spirogyra* sp., *Oedogonium* sp., *Caespitella* sp., etc.) and bacteria (*Flavobacterium* sp., *Crocinitomix* sp., *Undibacterium* sp., *Pseudomonas* sp., etc.) (Figure 3). Dominant bacteria in algal consortia are aerobic and non-pathogenic. The bacterial profile of the water sample was highly similar to that of algal consortia (Figure 3b). Dominant bacteria, such as *Flavobacterium* sp. and *Alpinimonas* sp., in the water sample are aerobic and non-pathogenic as well. Therefore, the utilization of endogenous algal consortia for AW treatment in aquaculture practice will not have pathogenic or toxic effects on aquatic animals.

The non-sterilized AW also contained a variety of bacteria, such as *Flavobacterium* sp., *Alpinimonas* sp., and *Crocinitomix* sp., which were co-existing with algae during AW treatment (Figure 3b). Accordingly, a synergistic relation between algae and bacteria could be developed based on the solid organic decomposition and CO_2_/O_2_ exchange (1, 22). By contrast, when the pure algae were inoculated in the sterilized AW, due to the lack of bacterial metabolisms, algal cells may not efficiently degrade solid organics in AW, resulting in the low biomass yield and TOC removal efficiency.

### 3.3 Roles of algae and wastewater-borne bacteria in AW remediation

In the laboratory-scale experiment, algae have different performances in sterilized AW and non-sterilized AW. As shown in Figure 4a, by the end of 10-day cultivation, biomass yield of pure algae grown in sterilized AW and algal consortia grown in non-sterilized AW reached 0.64 and 0.90 g/L, respectively. In addition, a higher removal efficiency of TOC (80.38%) was achieved by growing algal consortia in the non-sterilized AW. By the end of algae cultivation, residual concentrations of TOC in sterilized and non-sterilized samples were 109.6 and 46.8 mg/L, respectively (Figure 4b). Higher removal efficiencies of TN and TP in the non-sterilized AW were also observed (Figure 4c). Therefore, in a real-world application, it is algal consortia that should be employed for AW treatment, and it is not necessary to isolate the dominant algae from algal consortia (23).

It should be noted that the decomposition of SS in AW was improved by the co-growth of algae and bacteria. The content of SS dropped to 0.11 g/L in the non-sterilized AW, while that in the sterilized AW was 0.31 g/L (Figure 4c). Hence, in the AW treatment, the existence of bacteria promoted the decomposition of SS and released more nutrients from wastewater for algae growth. This result is in accordance with some previous studies, which reported the cooperation between algae and bacteria for nutrient removal in animal manure, food processing effluent, municipal wastewater, and so on (24–26). In fact, the direct use of non-sterilized AW not only favors the biomass production and nutrient removal, but also reduces the total cost of AW pretreatment. In the co-growth system, there are synergistic relations between algae and bacteria in O_2_/CO_2_ exchange and organic utilization. On one hand, algae absorb CO_2_ and release O_2_ through photosynthesis, while bacteria consume O_2_ and generate CO_2_ via heterotrophic metabolisms (27). In the micro-environment, the exchange of CO_2_ and O_2_ between algae and bacteria could be favorable to the growth of microorganisms and biomass production. On the other hand, extracellular enzymes secreted by bacteria promoted the conversion of SS to low-molecular-weight organics, enhancing the nutrient assimilation by algal cells (28, 29).

Figure 4d shows that, in terms of the contents of crude protein and crude lipid, there is no obvious difference between the harvested biomass from sterilized and non-sterilized AW. In addition, carbon content in the biomass of pure algae and algal consortia reached 49.49 and 50.21%, respectively. Accordingly, carbon retention by algal consortia growth in AW was 351.5 mg carbon/L wastewater. According to Equation 1, carbon emission of AW treatment by pure algae and algal consortia reached 20.74 and −112.97 mg carbon/L wastewater, respectively. Therefore, with the efficient assimilation of carbon by algal consortia, AW treatment became a negative carbon emission process. Compared with the bacteria-free condition, the existence of wastewater-borne bacteria was more favorable to the carbon sequestration of algae in the AW treatment. Under this situation, the treatment of 1 m^3^ non-sterilized AW by algal consortia is accompanied by the absorption of 0.414 kg CO_2_ (0.209 m^3^ CO_2_).

These results indicate that with the application of algae-based nutrient recovery, carbon emissions during the AW treatment could become negative. Such a promising phenomenon is mainly attributed to two facts, namely the direct assimilation of organic carbon in AW and the capture of atmospheric CO_2_ by photosynthesis. On one hand, the direct assimilation of organic carbon reduced the CO_2_ released from AW, lowering the carbon emission of AW treatment. On the other hand, the conversion of atmospheric CO_2_ to algae biomass compensates for the carbon emission of AW treatment. As a result, with the algae-based carbon assimilation, a carbon-negative AW treatment process is developed.

### 3.4 Enhancement of carbon sequestration by parameter optimization

**Figure 5:**
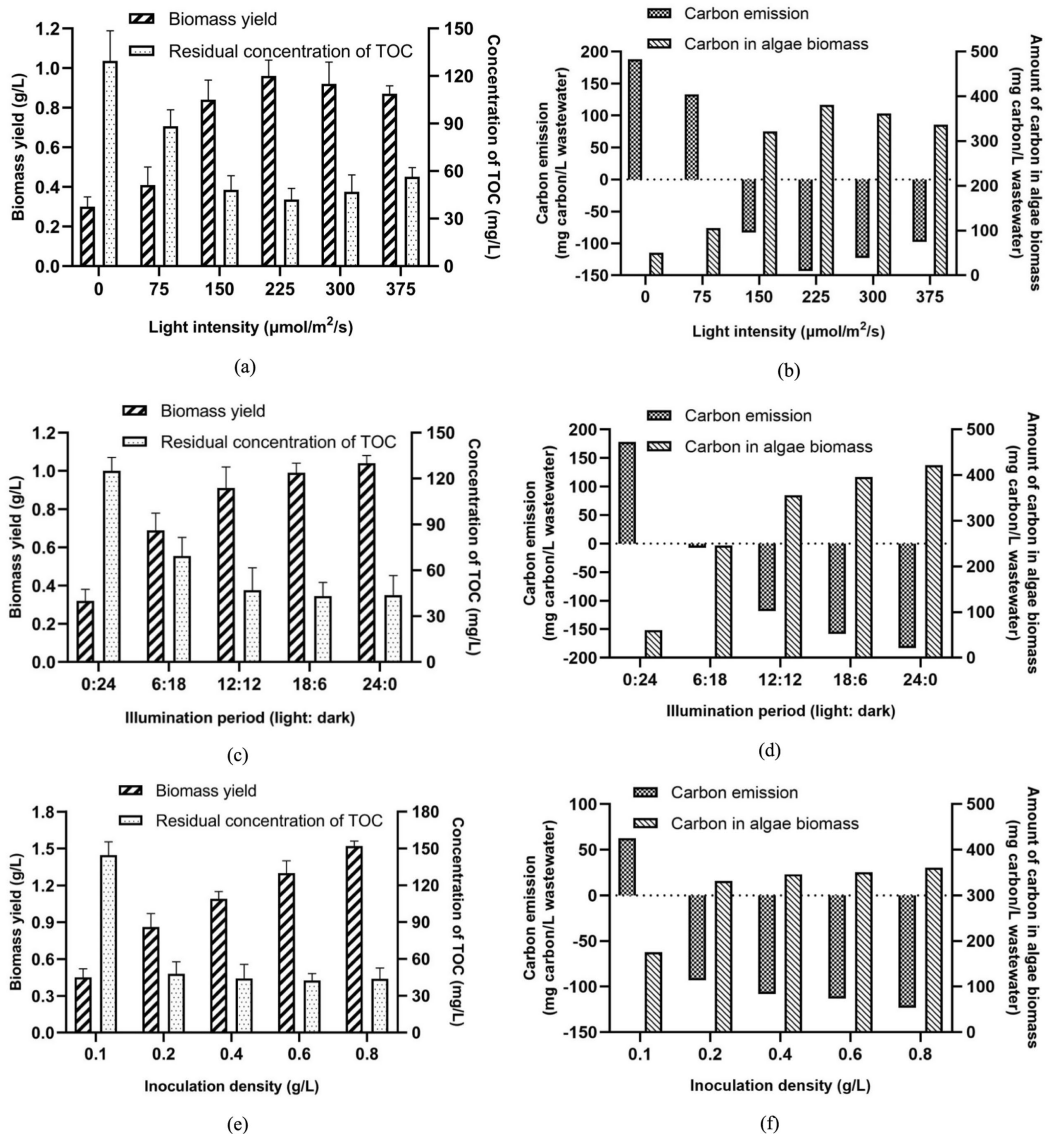
Effects of three major parameters on algae growth and carbon emission during AW treatment **(a)** Light intensity; **(b)** Illumination period; **(c)** Inoculation density of algae.

As shown in Figure 5a, biomass yield of algal consortia was improved with the increase of light intensity, reaching the peak value (0.96 g/L) when the light intensity was 225 μmol/m^2^/s. When the light intensity was 0 μmol/m^2^/s, the biomass yield of algal consortia was the lowest due to the inhibition of photosynthesis in the condition without illumination. Nevertheless, when the light intensity exceeded 225 μmol/m^2^/s, a slight drop of biomass yield was observed (Figure 5a). In addition, in terms of TOC removal, the optimal light intensity should be set as 225 μmol/m^2^/s since higher intensity did not further reduce the residual concentration of TOC. In a real-world application, the increase in light intensity is accompanied by an increase in electric energy consumption, so lower intensity is preferred for algae cultivation. Therefore, the optimal light intensity for algae cultivation for AW treatment was set as 225 μmol/m^2^/s. Excessively high light intensity can cause photosaturation, inhibiting the growth and metabolism of algal cells, and leading to increased energy consumption. Under this condition, the carbon emission of AW treatment was −185.20 mg carbon/L wastewater. Accordingly, the algae-based treatment of 1 m^3^ AW could result in the absorption of 0.679 kg CO_2_ (0.343 m^3^ CO_2_) (Figure 5b).

Figure 5c indicates that with the extension of the light period, the biomass yield of algal consortia increased gradually. It should be noted that when the light period was longer than 12 h each day, the extension of the light period did not further reduce the residual concentration of TOC. Hence, the improvement of biomass production was mainly attributed to the enhancement of photosynthesis of algae. According to the results in Figure 5c, the increase of the light period by 6 h (from 0 h to 6 h) improved the biomass yield by 0.37 g/L. By contrast, the increase of the light period by 6 h (from 12 h to 18 h) only improved the biomass yield by 0.17 g/L. In the view of the present authors, nutrient deficiency in AW may be the limiting factor of algae growth when the light period is long. Therefore, to save the electric energy consumption of the algae cultivation, the illumination period (light: dark) should be set as 12 h:12 h in this study. Under this situation, the carbon emission of AW treatment was −117.99 mg carbon/L wastewater Figure 5d, suggesting that 0.433 kg CO_2_ (0.219 m^3^ CO_2_) could be absorbed by the algae-based treatment of 1 m^3^ AW.

Inoculation density of algae is another factor that can influence the biomass yield and carbon sequestration in the AW treatment. When the inoculation density was 0.1 g/L, the biomass yield of algal consortia by the end of 10-day cultivation was only 0.45 g/L, resulting in high carbon emission (62.77 mg carbon/L wastewater) (Figures 5e,f). Due to the competition between wastewater-borne bacteria and algae for limited nutrients in AW, low inoculation density caused the lagged algae growth. Higher inoculation density of algal consortia would make algae the dominant species in the microbial community, accelerating the production of algae biomass. However, Figure 5e demonstrates that when the inoculation density of algae exceeded 0.2 g/L, no obvious drop in TOC concentration was observed. In addition, carbon emission was not dramatically reduced when the inoculation density of algae increased from 0.2 to 0.8 g/L. The main reason for this phenomenon is that high inoculation density of algae increased the turbidity of the transparency of the culture medium, making it difficult for light to reach some algal cells. This impaired algal photosynthesis, resulting in no significant improvement in the overall carbon absorption capacity of the algae culture process. Therefore, in this study, the inoculation density of algal consortia was set as 0.2 g/L.

Under the optimal conditions mentioned above, biomass yield of algal consortia, residual concentration of TOC in AW, and carbon emission during AW treatment reached 0.96 g/L, 42.1 mg/L, and −143.10 mg carbon/L wastewater (absorption of 0.525 kg CO_2_/m^3^ wastewater or 0.265 m^3^ CO_2_/m^3^ wastewater), respectively (Figures 5a,b). In this way, AW treatment based on algal consortia under the optimal conditions becomes a carbon-absorbing process. This can be regarded as a technological advancement in the in the field of carbon emission reduction in AW treatment.

Compared with aquaponics, in which vegetables could assimilate organic carbon and atmospheric CO_2_ (30), algal consortia-based AW remediation has obvious advantages, such as a higher carbon sequestration rate, shorter cultivation period of vegetables, and more efficient utilization of biomass (9, 31). First, photosynthetic algae have much higher photosynthetic efficiency per unit area than vegetables. Particularly, the carbon concentrating mechanism (CCM) improved the carbon bio-sequestration capacity of algae under the condition of low CO_2_ concentration. For example, in the study of Licamele (32), 434 plants (lettuce) were cultivated on a hydroponic bed (2.4 m × 4.8 m × 0.46 m) with a volume of 5,436 L for AW treatment and yielded vegetables with a mean dry weight of 4.36 g per plant (32). Under this situation, in the 35-day AW treatment, 1892.24 g of vegetable biomass (*dry weight*) was produced. However, according to the data in the present study, the biomass yield of algal consortia reached 0.96 g/L. In the pond with the same volume (5,436 L), 5218.56 g algae biomass (*dry weight*) could be produced in the 10-day AW treatment. It was reported that carbon sequestration in plants of aquaponics could only offset 40 to 62% of direct greenhouse gas emissions (33). By contrast, in the present study, carbon sequestration of algal consortia even exceeded the carbon input of the whole aquaculture system. Second, the cultivation periods of algal consortia and algae are much shorter than those of hydroponic vegetables. According to previous studies, the cultivation periods of commercial vegetables in aquaponics could reach 35 days, 197 days, 44 days, and 90 days, respectively (32, 34–36). By contrast, the cultivation periods of algae and algal consortia in AW could be controlled around 10 days (37, 38). Third, the harvested vegetable might deteriorate easily, while algae biomass can be stored for a long period of time. It was estimated that in Pakistan, 35–40% of vegetables and fruits were wasted after harvesting due to the severe losses and deterioration (39). Munhuewyi (40) reported that up to one-third of all fresh vegetables (about 1.3 billion tonnes) are lost along the post-harvest supply chain and never reach the consumers (40). The deterioration of a large amount of fresh vegetables is accompanied by intensive CO_2_ emission, indirectly lowering the carbon retention capacity of the aquaponic system. By contrast, in practice, the harvested algae biomass is normally dehydrated to produce dry algae powder, which is further used as feedstock for algae oil extraction, animal feed production, or food production, attenuating the biomass deterioration and preventing carbon emission.

### 3.5 Long-term operation of the pilot-scale aquaculture system

**Figure 6:**
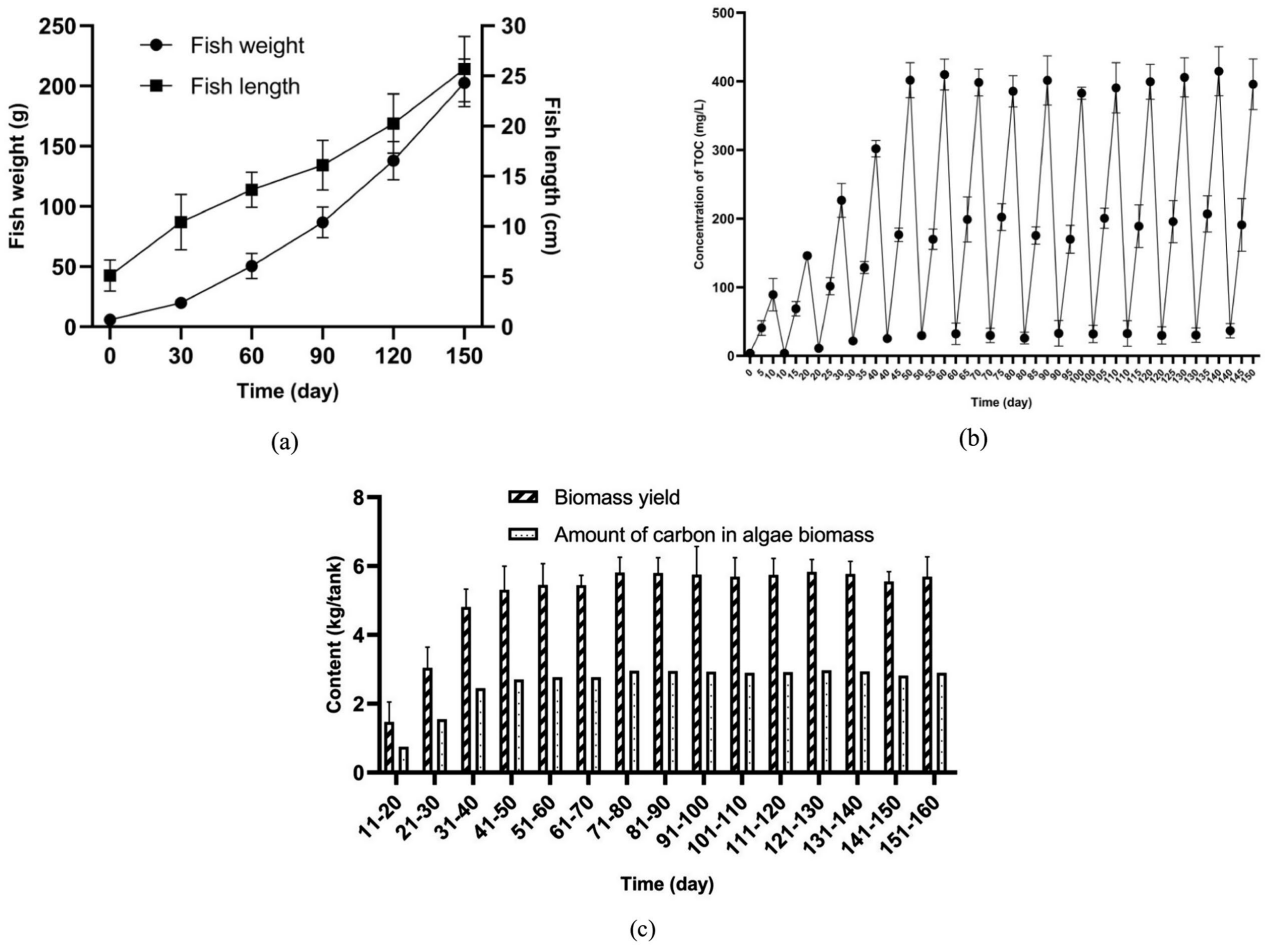
Long-term operation of the pilot-scale aquaculture system **(a)** Growth of fish; **(b)** Concentration of TOC in fish-rearing tank; **(c)** Biomass yield of algal consortia.

As shown in Supplementary Table S1, in the 150-day period, with the growth of fish, the average amount of feed added for fish rearing increased from 140 to 910 g/tank/day gradually. Accordingly, the daily carbon input of the fish-rearing tank was improved from 71.68 to 465.92 g/tank/day. Due to the continuous input of feed in the fish-rearing tank, the fast growth of fish was observed during the 150-day experiment. Figure 6a shows that through the 150-day culture, the mean weight of largemouth bass increased from 5.84 to 202.62 g gradually. Moreover, the mean length of fish reached 20.68 cm by the end of the 150-day experiment. It should be noted that only a portion of the feed added for fish rearing was converted to fish meat, while the other portion of the feed became fish feces, which can be regarded as a major pollution source in the aquaculture system.

In this experiment, due to the accumulation of fish feces, the concentration of TOC in water increased (Figure 6b). For example, in the period of Day 0 to Day 10, the concentration of TOC in the water of the fish-rearing tank increased from 3.85 to 89.10 mg/L. It is noteworthy that in the later stage (Day 60–150) of the 150-day experiment, a higher concentration of TOC (around 400 mg/L TOC) in the fish-rearing tank was detected before the water recycling. This phenomenon is mainly attributed to the larger amount of feed added for fish rearing in the later stage. For example, Figure 6b shows that the increase of TOC concentration in the fish-rearing tank before the water recycling during the periods of Day 0–10, Day 30–40, Day 60–70, Day 90–100, and Day 120–130 reached 85.25, 280.09, 366.11, 349.90 and 375.97 mg/L, respectively. As shown in Supplementary Table S2, during the corresponding periods, total carbon inputs over 10 days were 0.72, 2.51, 3.64, 4.17, and 4.51 kg/tank, respectively. In the experiment, increasing the amount of feed added to the fish-rearing tanks resulted in greater production of fish feces and feed residues. A portion of this waste settled at the bottom of the tank, while the remainder dissolved into the water.

When water enriched with fish feces and feed residues enters the sedimentation tanks and algae cultivation pond, it promotes algal growth. Figure 6c indicates that the amount of algae biomass harvested from the aquaculture system increased gradually with the enrichment of TOC in water. For instance, the amount of the harvested algae biomass in Day 10–20 was only 1.47 kg, while that in Day 150–160 reached 5.69 kg. At the same time, with the assimilation of organics in water by algal consortia, the concentration of TOC dropped (Figure 6b).

**Figure 7:**
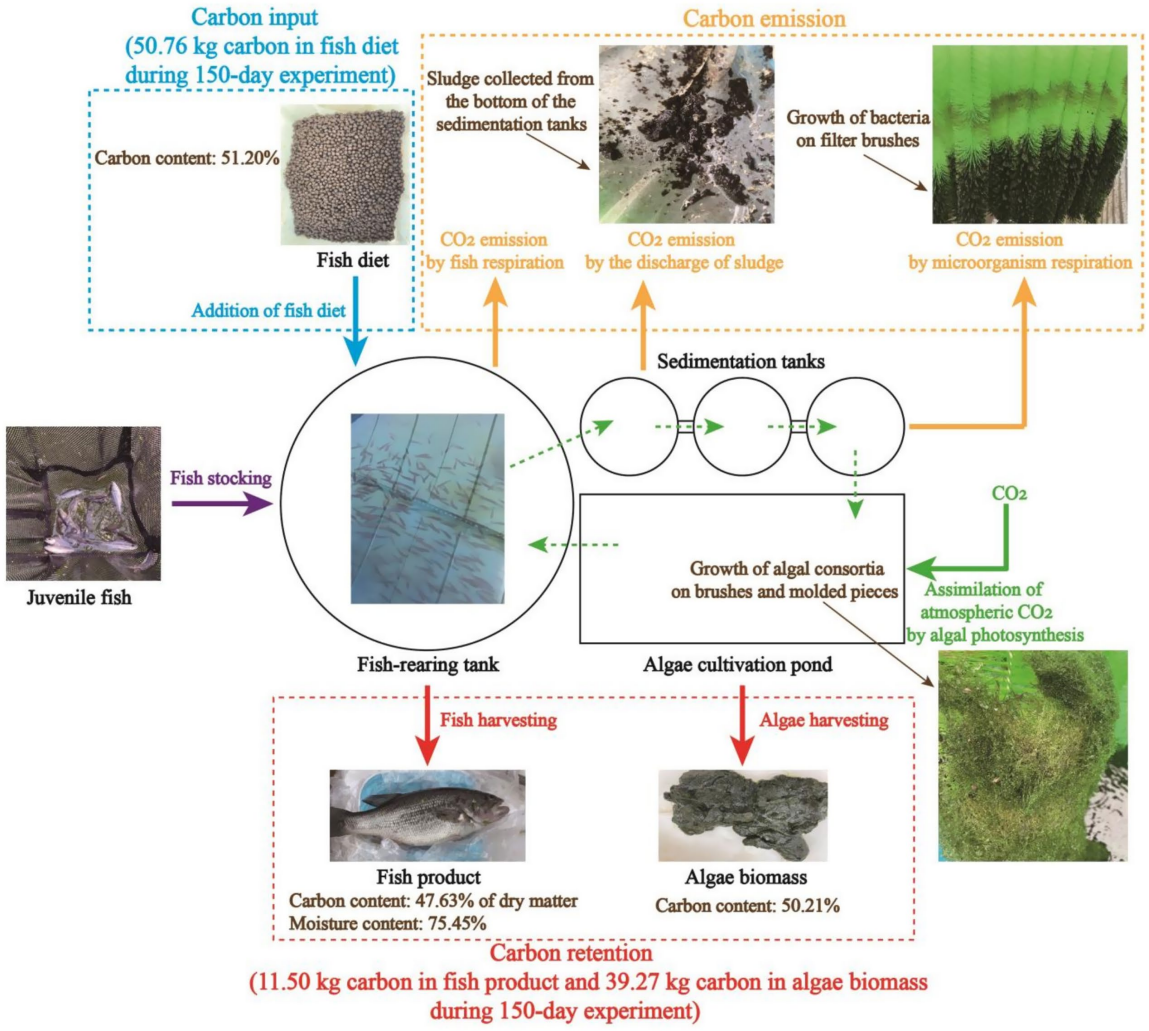
Carbon flux in the long-term operation of the pilot-scale aquaculture system.

As shown in Figure 7, total carbon input throughout the whole 150-day experiment was 50.76 kg, and 11.50 kg of carbon was outputted as fish product. At the same time, 77.15 kg of algae biomass, which contained 39.27 kg of carbon in total, was produced. This result demonstrates that carbon output in the form of fish and algae biomass was slightly higher than carbon input, making the fish-rearing activity in this recirculating aquaculture system a net-zero carbon process. Particularly, the footprint of the algae cultivation system was only 6.12 m^2^, increasing the practical applicability of the algae-based AW treatment for carbon sequestration. In this experiment, it should be noted that not all of the organic carbon in the feed was converted to fish meat or assimilated by algal consortia. In fact, a portion of carbon in the feed was converted to CO_2_ through the respiration of fish and the metabolism of heterotrophic microorganisms. In addition, a certain amount of carbon was retained at the bottom of the sedimentation tanks in the form of sludge (Figure 7). Fortunately, algal consortia could sequestrate atmospheric CO_2_ via photosynthesis, thus compensating for the CO_2_ released from the aquaculture system. As a result, due to the excellent performance of algal consortia in carbon sequestration, a net-zero carbon process was developed for fish-rearing and AW treatment in a recirculating aquaculture system.

As shown in Table 3, the survival ratio of the fish during the whole pilot-scale experiment was 99.4%, suggesting that the AW treated by algal consortia could be recycled for fish rearing without any obvious toxic effect on fish growth. In addition, the cost of microalgae harvesting may account for approximately 30% of the total cost of microalgae production (30), while the dominant algae used in this study were filamentous algae, which provided structural support for the formation of algal consortia (41). Compared with unicellular microalgae, such as *Chlorella* sp. and *Scenedesmus* sp., endogenous algal consortia used in this study could have much lower production costs due to the simplification of the harvesting process. Finally, the harvested algae biomass can be used as feed for shellfish, which can partially convert the carbon into CaCO_3_ in their shells. It has been widely recognized that the carbon in shells and the carbon that enters sediments through biodeposition are long-lived forms of carbon (42). Hence, the use of algae biomass harvested from AW for shellfish farming can be regarded as a promising way toward the permanent storage of organic carbon in AW (42).

**Table 3:** Growth, feed utilization, and survival of fish in the 150-day pilot-scale experiment, and the major composition of fish.

Growth parameters	Value	Major compositions of fish	Value
Initial weight (g)	5.84	Content of protein (%) in fish	18.93%
Final weight (g)	202.62	Content of lipid (%) in fish	3.27%
Weight gain (g)	196.78	Moisture content (%) in fish	75.45%
Initial length (cm)	5.11	Carbon content (%) in the dry matter of fish	47.63%
Final length (cm)	20.68		
Length increase (cm)	15.57		
Percent weight gain (%)	3369.52%		
FCR^a^	1.01		
PER^b^	2.32		
Survival ratio (%)	99.4%		

a Feed conversion ratio.

b Protein efficiency ratio.

## 4 Conclusion

This study developed a novel method of employing endogenous algal consortia for AW remediation. It was discovered that endogenous algal consortia, of which *Schizomeris* sp. was the dominant genus, performed well in the nutrient removal and carbon assimilation for AW treatment. Under the optimal conditions in laboratory research, algae-based AW did not release CO_2_, but became a carbon-absorbing process. In the pilot-scale experiment, with the algae-based carbon sequestration, a net-zero carbon process was developed for fish-rearing and AW treatment.

In the practical implications, the results of this study could be adopted to improve the carbon neutrality, economic viability, and ecological resilience of aquaculture activity. First, the intensive carbon emission of AW treatment could be reduced by the algae-based carbon absorption, achieving a net-zero carbon emission aquaculture system. Second, filamentous algae enable low-cost biomass harvesting, and protein-rich algal biomass serves as a high-value feed ingredient, increasing the total profitability of AW treatment. Third, endogenous consortia thrive in aquaculture conditions without pathogen risks, ensuring system stability and water recycling safety.

## Data Availability

The original contributions presented in the study are included in the article/Supplementary material, further inquiries can be directed to the corresponding authors.
