# Supplementary material for: Cultivation of endogenous algal consortia for aquaculture wastewater remediation as a strategy toward net-zero carbon emission: from laboratory research to practical application
Source: Front Nutr. 2025 Sep 8;12:1600232. doi: 10.3389/fnut.2025.1600232 (PMC12450980; doi:10.3389/fnut.2025.1600232)
Supplement: Supplementary file 1 [file Data_Sheet_1.pdf]

Table S1. Actual levels of the independent variables for the experiment of single-factor experiment

No.	Independent variables		
	Light intensity (μmol/s/m^2)	Illumination period (light:dark)	Inoculation density of algae (g/L)
1	0, 75, 150, 225, 300, 375	12:12	0.2
2	150	0:24, 6:18, 12:12, 18:6, 24:0	0.2
3	150	12:12	0.1, 0.2, 0.4, 0.6, 0.8

Table S2. Average amount of feed added for fish-rearing

Period	Average amount of fish feed (g/tank/day) for each fish-rearing tank	Average amount of fish feed (g/fish/day) for each fish	Daily carbon input (g/tank/day) in each fish-rearing tank	Total carbon input (kg/tank) in each fish-rearing tank during 10-day period
Day 0-10	140	0.28	71.68	0.72
Day 10-20	235	0.47	120.32	1.20
Day 20-30	370	0.74	189.44	1.89
Day 30-40	490	0.98	250.88	2.51
Day 40-50	580	1.16	296.96	2.97
Day 50-60	625	1.25	320.00	3.20
Day 60-70	710	1.42	363.52	3.64
Day 70-80	750	1.50	384.00	3.84
Day 80-90	790	1.58	404.48	4.04
Day 90-100	815	1.63	417.28	4.17
Day 100-110	850	1.70	435.2	4.35
Day 110-120	880	1.76	450.56	4.51
Day 120-130	880	1.76	450.56	4.51
Day 130-140	890	1.78	455.68	4.56
Day 140-150	910	1.82	465.92	4.66

Figure caption

Figure S1. Pictures of endogenous algal consortia (a. Attachment of algal consortia on wall surface; b. Harvest of algal consortia from algae cultivation pond)

Figure S2. Removal of TOC and SS in AW by Fenton reaction

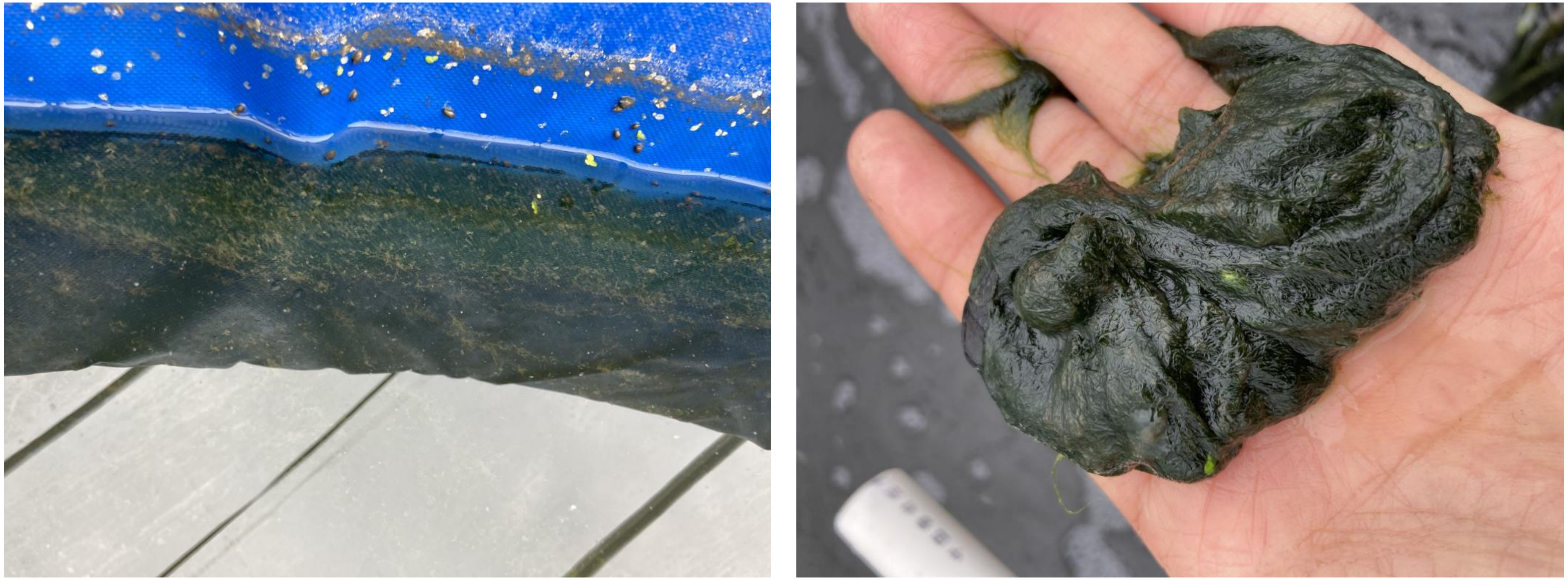

(a) (b)

Figure S1

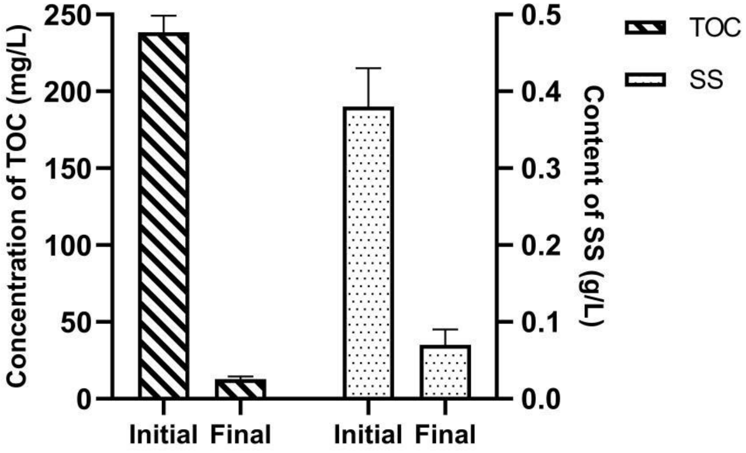
Concentration of TOC (mg/L)
250
200
150
100
50
0
Content of SS (g/L)
0.5
0.4
0.3
0.2
0.1
0.0
Initial
Final
Initial
Final
TOC
SS

Figure S2
